# Editorial: Targeting Phosphorylation and Glycosylation of Membrane-Bound Oncogenic Drivers, Receptors and Adhesion Molecules to Control Constitutive Proliferation of Human Carcinoma Cells

**DOI:** 10.3389/fonc.2018.00283

**Published:** 2018-07-31

**Authors:** Muhammad R. M. Hussain, Daniel C. Hoessli

**Affiliations:** ^1^CAS Key Laboratory of Genomic Sciences and Information, Beijing Institute of Genomics, Beijing, China; ^2^University of Chinese Academy of Sciences, Beijing, China; ^3^Panjwani Center for Molecular Medicine and Drug Research, International Center for Chemical and Biological Sciences, University of Karachi, Karachi, Pakistan

**Keywords:** glycosylation, phosphorylation, cancer, sialylation, fucosylation

Surface proteins in cancer cells are extensively modified with *N*- and *O*-glycans, bearing anomeric features that are distinct from those found associated with the corresponding gene products in non-neoplastic cells. From GWAS studies, abundant clues point to glycosyltransferases as generators of glycan diversity in neoplastic cells. In addition, glycans may influence the conformations of O- and N-glycosilation of cell membrane glycoproteins in cancer cells, thereby modifying their allosteric properties in signaling, and altering their binding to cell extracellular agonists, antibodies, and other defense proteins. For instance, glycosylation of the extracellular portions of EGFR controls the allosteric conformation of intracellular portions of the transmembrane EGFR and, most importantly, its tyrosine kinase catalytic activity, which initiates pathways leading to uncontrolled proliferation and resistance to apoptosis in cancer cells. From a structural point of view, the glycan patterns exhibited by neoplastic glycoproteins are recognition sites to which innate and acquired defense proteins bind and determine the fate of abnormally growing cells in a given organism. Our topic “Targeting Phosphorylation and Glycosylation of Membrane-Bound Oncogenic Drivers, Receptors and Adhesion Molecules to Control Constitutive Proliferation of Human Carcinoma Cells” discusses such features of glycobiology, comprising two original articles, one review, and one perspective article.

As the charge and polarity environment are the key features of protein conformation in both ER and Golgi, the original article by Manwar Hussain et al. discusses how particular patterns of charge and polarity amino acids surrounding Asn may provide the favorable environment for *N*-glycosylation (Manwar Hussain et al.). The authors carried out genome-wide mapping of 1,117 proteins for drawing the relationship between *N*-glycosylation and neoplastic phenotypes. EGFR and TGFB1, and E-cad were selected for highlighting the correlation of charge and polarity with aberrant glycosylation.

In their original article, Möginger et al. show that, by utilizing state-of-the-art mass spectrometry technologies, the human skin *N*-glycome contains a much larger proportion of complex glycans than the *O*-glycome (Möginger et al.). They further determined that sequences of oligomannose type *N*-glycans are predominantly expressed in basal cell carcinoma (BCC) and squamous cell carcinoma (SCC), as compared to normal skin, and the glycopeptide analyses revealed that the glycoproteins carrying such glycan motifs were mostly found in glycoproteins involved in binding events.

In their review article, Blanas et al. elaborate on the role of fucosylated glycans in cancer and its prognosis (Blanas et al.). The authors review the fucosylated epitopes, particularly the Lewis antigens and their contribution to cancer cells proliferation, invasion, metastatisation, interaction with immunocytes and endothelial cells, and finally how they may promote resistance to therapy.

In their perspective article, Haas et al. cover the involvement of Siglecs (Siglecs) and sialyltransferases (STs) in breast, ovarian, and uterine cancers (Haas et al.). As hypersialylation is a known cancer marker, defining the balance between sialosides and sialic acid-binding Ig-like lectins is a valid approach to understanding antitumor immunity.

In summary, the role of glycosylation in cancer cell heterogeneity is becoming one major challenge to unravel the mechanisms whereby neoplastic cells interact with their cellular environment in invasion and metastasis, as well as interactions with immune and non-immune defense mechanisms. As aberrant glycosylation is now recognized as a cancer hallmark (1), ways to tamper with expression and function of cancer-specific glycans is attracting legitimate attention in anti-cancer therapy. Likewise, the realization that extracellular glycosylation motifs of transmembrane tyrosine kinases also conditions the catalytic activity of intracellular kinases makes proliferation motors like EGFR dependent upon the nature and amounts of their glycan moieties and invites glycan blocking or inhibiting strategies as ways to interfere with proliferation and apoptosis resistance.

## Author contributions

All authors listed have made a substantial, direct and intellectual contribution to the work, and approved it for publication.

### Conflict of interest statement

The authors declare that the research was conducted in the absence of any commercial or financial relationships that could be construed as a potential conflict of interest.
